# Reoperations for complications within 90 days after gel stent implantation or trabeculectomy

**DOI:** 10.1007/s10792-022-02575-0

**Published:** 2022-11-23

**Authors:** Carlo A. Cutolo, Chiara Bonzano, Carlo Catti, Chiara Pizzorno, Alessandro Bagnis, Carlo E. Traverso, Michele Iester

**Affiliations:** 1 DiNOGMI, Clinica Oculistica, University of Genoa, Genoa, Italy; 2 IRCCS Ospedale Policlinico San Martino, Genoa, Italy

**Keywords:** Glaucoma surgery, Complications, Clinical outcome, Trabeculectomy, Gel stent

## Abstract

**Purpose:**

To describe reoperations in the operating room for complications that occurred within the first 90 days after gel stent implantation or trabeculectomy at a single institution over 5 years.

**Methods:**

In this retrospective chart review, patients who have undergone gel stent implantation with mitomycin C (MMC) or trabeculectomy with MMC were enrolled. Postoperative complications that required reoperations within the first 90 days were evaluated.

**Results:**

A total of 510 surgeries were performed on 392 patients over a 57-month period by 2 glaucoma surgeons. Of these, 284 were gel stent implantation, and 226 were trabeculectomy. Combined phacoemulsification was performed in 52/284 (18.3%) in the gel stent group and in 26/226 (11.5%) of eyes in the trabeculectomy group (*p* = 0.03). Reoperations took place in 13/510 (2.5%) eyes, including 4/284 (1.4%) in the gel stent group, 9/226 (4.0%) in the trabeculectomy group (*p* = 0.07). In the gel stent group, indications for reoperation were bleb failure (2), suprachoroidal hemorrhage (1), bullous keratopathy (1). In the trabeculectomy group, indications for reoperation were bleb failure (3), overfiltration (2), persistent wound leak (2), aqueous misdirection (2).

**Conclusions:**

The rates of reoperation for early postoperative complications after gel stent or trabeculectomy was low and comparable with previous studies. A slightly higher number of reoperations within 90 days was observed in the trabeculectomy group than the gel stent group despite the more significant number of combined procedures in the latter group. Bleb failure was the most common indications for reoperation in both groups. Excessive outflow was a cause of reoperation mostly in the trabeculectomy group.

## Introduction

Trabeculectomy remains the most performed glaucoma surgery across the world because of its well-established long-term efficacy [1]. However, trabeculectomy procedure may be associated to several serious intraoperative and perioperative complications that may require unplanned reoperations [1–3]. Alternative methods to achieve sub-conjunctival filtration have been progressively introduced in the clinical practice with the aim to increase the safety of the filtering procedure [4]. Among them, gel stent is a 6-mm gelatin tube with a 45-µm lumen size designed to be implanted ab-interno and that deliver humor aqueous from the anterior chamber to the subconjunctival space [5]. The gel stent implant procedure is deemed to achieve subconjunctival filtration with fewer periprocedural complications than trabeculectomy [6]. When considering the first 90-day, Chu et al. found that reoperations for complications within 90 days after trabeculectomy were 2.5%. In another recent study, Shalaby et al. [7, 8] found reoperation within 90 days after gel stent to be up to 10.5%. This observations are apparently in contrast with a large retrospective study that compared the efficacy and safety of gel stent and trabeculectomy and found a reoperation rate of 5.3% and 10.3%, respectively [9]. Unplanned reoperations place considerable burden on patients recovering from glaucoma surgery and reoperation rate is an important determinant of procedural efficacy and safety [10]. An unplanned return to the operating room may represent a surrogate of problems related to the procedure itself and the 90 days reoperation rate has not yet been directly compared between trabeculectomy and gel stent implant. Estimating the number and causes of complications is meant to inform the patients better and offer the best therapeutic choice for each eye. The aim of our study was to describe reoperations in the operating room for complications that occurred within the first 90 days after gel stent implantation or trabeculectomy.

## Methods

This retrospective study was carried out at Clinica Oculistica, Genova, Italy. The electronic health records (EHR) system was queried for adult patients that underwent trabeculectomy or gel stent implantation over 54 months, starting March 1, 2016, to September 31, 2020. Standalone and combined glaucoma procedures with phacoemulsification and intraocular lens implantation (IOL) were also included in the analysis. The starting date was chosen because it was the date when the gel stent was firstly used in the clinical practice at our centre. Then, the EHR records, exported into an Excel file, were filtered for identifying patients that had additional surgery in the same eye within 90-day period and the observation time was extended till December 31, 2020. All the identified records were then checked for any mistake and a definitive list of early reoperations was created. Both eyes of a patient were included in the analysis if they met the criteria. Primary outcome was the number and causes of unplanned reoperations defined as any ocular condition requiring a return to the operating room within a 90-day period. Interventions performed at the slit lamp, such as needling procedures, suture lysis, and anterior chamber reformation, postoperative antimetabolites subconjunctival injection were not considered as reinterventions. Data abstracted from medical records included patient demographics, glaucoma subtype, lens status, previous intraocular surgeries, IOP, number of glaucoma medications, glaucoma stage, type of glaucoma surgery, the time interval between reoperation, and type of surgical complication requiring reoperation. Two skilled glaucoma surgeons (C.E.T, M.I.) performed all the procedures analyzed (53% CET, 47% M.I). Based on previous research [7, 8], our study was powered at 95% to detect the predicted difference in the primary outcome. Fisher exact test was used to compare categorical variables among the 2 groups, including the rate of combined procedures and the rate of reoperations. A *p*-value of < 0.05 was considered statistically significant. All analyses were conducted in Stara 15.1 (StataCorp LLC, College Station, TX).

## Results

**Fig. 1:**
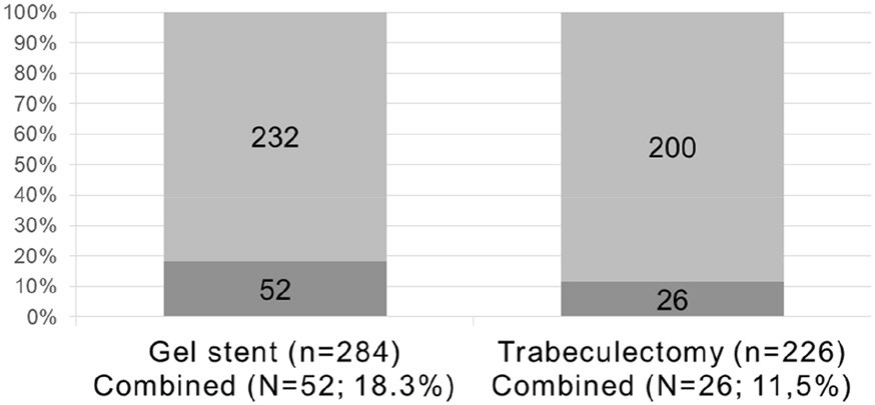
Proportions of standalone and combined procedures

Based on inclusion criteria, 510 surgeries performed on 392 patients were filtered for the analysis. Namely, 284 (55.7%) were gel stent implantation, and 226 (44.3%) were trabeculectomy. The glaucoma procedures were combined with phacoemulsification-IOL implantation in 52/284 (18.3%) cases and 26/226 (11.5%) cases among the gel stent group and the trabeculectomy, respectively (*p* = 0.03; Fig. 1). Unplanned reoperations within 90-day period took place in 13/510 (2.5%) eyes, including 4/284 (1.4%) in the gel stent group, 9/226 (4.0%) in the trabeculectomy group (*p* = 0.07). Table 1 summarizes the demographic and ocular characteristics of patients included in the study whereas Table 2 summarizes the demographic and ocular characteristics of patients and eyes that underwent reoperation. Table 3 reports the causes of reoperation. Bleb scarring was observed as a cause of reoperation for both groups, whereas bleb leaking and over filtration were only observed in the trabeculectomy group. Two cases of aqueous misdirection that required early reoperation were only observed in the trabeculectomy and occurred in eyes with the previous diagnosis of angle-closure glaucoma. The case of suprachoroidal haemorrhage occurred in an eye with a previous diagnosis of post-traumatic glaucoma that underwent pars-plana vitrectomy 2 years before the gel stent implantation. In no case, both eyes of the same patient experienced early reoperation. The mean number of days elapsed between the glaucoma procedure and reoperation was 39.5 ± 34.2 and 38.2 ± 18.7 for the gel stent and trabeculectomy, respectively (*p* = 0.95).

**Table 1:** Demographic and ocular characteristics

	Gel stent	Trabeculectomy	*P*-value
Total number of patients	219	173	
Age, years	70.4 ± 13.3	69.0 ± 11.2	0.25*
Gender, female	115/219	94/173	0.76**
Total number of eyes	284	226	
Glaucoma type			0.42**
POAG	218	163	
PACG	11	15	
PXFG	43	31	
JOAG	3	5	
Post-traumatic	3	4	
Other	6	8	
Lens status			0.13**
Phakic	226	164	
Pseudophakic	56	58	
Aphakia	2	4	
Preoperative IOP	24.3 ± 6.1	25.2 ± 4.7	0.07*
Glaucoma stage			0.08**
Early	36	15	
Moderate	154	130	
Advance	94	81	

*POAG* primary open-angle glaucoma, *PACG* primary angle-closure glaucoma, *PXFG* pseudoexfoliative glaucoma, *JOAG* jouvenile open-angle glaucoma, *IOP* intra-ocular pressure

^*^t-test

^**^chi-square test

**Table 2:** Demographic and ocular characteristics of cases that that required reoperation within 90 days

	Gel stent	Trabeculectomy
Total number of patients	4	9
Age, years	65.0 ± 14.6	57.2 ± 20.3
Gender, female	2/4	4/9
Glaucoma type		
POAG	2	4
PACG	0	2
PXFG	1	2
JOAG	0	1
Post-traumatic	1	0
Lens status		
Phakic	1	6
Pseudophakic	2	3
Aphakia	1	0
Preoperative IOP	26.5 ± 5.2	27.2 ± 4.7
Preoperative IOP-lowering medication, n	3.1	2.7
Glaucoma stage		
Early	0	0
Moderate	2	4
Advance	2	5

*POAG* primary open-angle glaucoma, *PACG* primary angle-closure glaucoma, *PXFG* pseudoexfoliative glaucoma, *JOAG* jouvenile open-angle glaucoma, *IOP* intra-ocular pressure

**Table 3:** Count of postoperative complications that required reoperation within 90 days

	Gel stent	Trabeculectomy
Bleb scarring	2	3
Bleb leak	0	2
Acqueous misdirection	0	2
Suprachoroidal hemorrage	1	0
Overfiltration and hypotony maculopathy	0	2
Corneal decompensation	1	0

A statistically significant difference between the proportion of combined procedures was observed between the two groups (*p* = 0.03).

## Discussion

In our study, we reported the rate and the causes of unattended reoperations that occurred within 90 days of trabeculectomy or gel stent placement at a single tertiary care institution. Among the 510 surgeries performed, postoperative complications requiring reoperations within 90 days occurred in 13 cases resulting in an overall reoperation rate of 2.4%. Although our research was powered enough to detect a difference between the two groups, the calculated difference was not significant (*p* = 0.07). Nevertheless, it is clinically meaningful to consider that the reoperation rate for trabeculectomy rate was almost three times the gel stent's reoperation rate despite the more significant number of combined procedures in the latter group. In the clinical practice, gel stent is combined with phacoemulsification more frequently than trabeculectomy and our study reflects this trend [6]. In a recent study, Chu et al. investigated the reoperation rate within 90 days after different glaucoma surgeries [7]. Chu et al. reported a reoperation rate of 2.5% for trabeculectomy, whereas, in our study, we found a higher reoperation rate for trabeculectomy (4.0%). However, Chu et al. focused their reports on unplanned reoperations, excluding reoperations for further IOP lowering. Performing the analysis using the same criteria as Chu et al., would obtain a similar 90-day reoperation rate of 2.7%. In our study, other causes of reoperation were mainly due to low IOP: overfiltration and wound leak. In Chu et al. study, wound leak was the leading cause of reoperation that occurred in 5 out of 7 cases. In our series of gel stent, wound leak was not an observed complication because we always opted for the ab-interno approach but may potentially occur if an ab-externo approach is performed [11].

When considering the gel stent group, we found a reoperation rate within 90 days of 1.4%. Recently, Shalaby et al. [12] reported the real-world reoperation rate within 90 days after gel stent implantation of 10.5%. The leading cause of reoperation described by Shalaby et al. was the needling procedure due to elevated IOP. In our study, needling procedures were mainly performed at the slit lamp as previously described. If we exclude needling, the reoperation rate reported by Shalaby et al. reduced to 3.9%.

In a recent study, Cardakli et al. analyzed the unplanned return to operating room rate time after trabeculectomy and the associated factors. A reoperation rate within 180 days of 9.5% was reported. When excluding bleb needling performed at the operating room, the reoperation rate was 6.5% at 180 days [3]. In the Cardakli study, leading causes of reoperation were bleb revision (32%), bleb needling (29%) and bleb revision with choroidal drainage (10%) and choroidal drainage without bleb revision (11%). In our study, choroidal drainage was performed in only one case of suprachoroidal haemorrhage that occurred in a patient with post-traumatic glaucoma and a previous pars-plana vitrectomy treated by gel stent implantation. In a previous study, we found that choroidal detachment was observed in 20% of cases after gel stent implantation, but it is an uncommon cause of reoperation [13, 14].

In the tube versus trabeculectomy study, the incidence and outcomes of reoperations for glaucoma after trabeculectomy or tube shunt implantation were investigated. In this multicenter randomized clinical trial, a higher rate of reoperation for glaucoma was found following trabeculectomy (29%) with mitomycin C than tube shunt surgery (9%) over a 5-year follow-up. These results are not comparable with ours because of the different follow-up considered and because the tube shunt surgical approach is very different compared to the gel stent implantation [15]. Our study is limited by the retrospective design and by the fact that early reoperations may have occurred elsewhere and not recorded in the database analyzed. This latter hypothesis is unlikely because our institution is a regional referral centre for glaucoma surgery. The strength of our study is that based on the results of previous studies, our number of cases analyzed resulted powered enough to detect a difference between the two procedures. In conclusion, the reoperation rate was slightly higher after trabeculectomy than after gel stent and bleb failure was the most common cause of reoperation within 90 days after trabeculectomy or gel stent implantation. Overfiltration and bleb leak were only observed in the trabeculectomy group.
